# Sedentary time at school and work in Canada

**DOI:** 10.17269/s41997-023-00835-9

**Published:** 2024-01-26

**Authors:** Stephanie A. Prince, Justin J. Lang, Marisol Betancourt, Stephanie Toigo, Karen C. Roberts

**Affiliations:** 1 Centre for Surveillance and Applied Research, Public Health Agency of Canada, Ottawa, ON Canada; 2 School of Epidemiology and Public Health, Faculty of Medicine, University of Ottawa, Ottawa, ON Canada; 3 Alliance for Research in Exercise, Nutrition and Activity (ARENA), University of South Australia, Adelaide, SA Australia

**Keywords:** Sedentary behaviour, School, Work, Surveillance, Adults, Youth, Sédentarité, école, travail, surveillance, adultes, jeunes

## Abstract

**Objectives:**

High levels of sedentary time (ST) are associated with poor physical and mental health. Given that Canadians spend a large portion of their days at school and work, they may be important targets for reducing ST. Our objectives are to estimate the daily amount of school and work ST among Canadians, examine differences by subgroups, and determine associations with health.

**Methods:**

Using the 2020 Canadian Community Health Survey Healthy Living Rapid Response module (*N* = 5242), the amount of time spent sitting while at school and work was estimated among youth (12–17 years) and adults (18–34 and 35–64 years). Differences by sociodemographics and 24-Hour Movement Guideline adherence were assessed with independent *t*-tests. Associations between school and work ST and health indicators were assessed using adjusted logistic regression.

**Results:**

Canadian youth aged 12–17 years and adults aged 18–34 years reported an average of 4.5 and 5.2 h/day of school ST, respectively. Adults 18–34 years and 35–64 years reported an average of 3.9 and 4.0 h/day of work ST, respectively. School and work ST differed within several subgroups. Among adults 18–34 years, higher school ST was associated with a reduced odds of ‘excellent/very good’ mental health, whereas higher work ST was associated with a greater likelihood of reporting ‘excellent/very good’ general health.

**Conclusion:**

Canadian youth and working-age adults report an average of 4–5 h/day sedentary at school or work. This is the first study estimating school and work ST in a representative sample of Canadians and will aid in increasing awareness of setting-specific behaviours to better inform targeted interventions including addressing inequalities in ST.

**Supplementary Information:**

The online version contains supplementary material available at 10.17269/s41997-023-00835-9.

## Résumé

**Objectifs:**

Des niveaux élevés de sédentarité sont associés à une mauvaise santé physique et mentale. Étant donné que les Canadiens passent une grande partie de leur journée à l’école et au travail, ils peuvent représenter des cibles privilégiées sur la question de la sédentarité. Nos objectifs sont les suivants : estimer le temps que les Canadiens passent assis par jour à l’école et au travail, c’est-à-dire le temps de sédentarité, examiner les différences qui existent entre les sous-groupes et déterminer les effets sur la santé.

**Méthodes:**

À l’aide du module de réponse rapide concernant un mode de vie sain de l’Enquête sur la santé dans les collectivités canadiennes de 2020 (N = 5 242), nous avons estimé le temps passé assis à l’école et au travail chez les jeunes (12–17 ans) et chez les adultes (18–34 ans et 35–64 ans). Nous avons évalué les différences en fonction des données sociodémographiques et en fonction du respect (ou non) des directives en matière de mouvement sur 24 heures, ce à l’aide de tests indépendants. Grâce à une régression logistique ajustée, nous avons évalué le lien entre la sédentarité à l’école et au travail et les indicateurs de santé.

**Résultats:**

Les jeunes Canadiens âgés de 12 à 17 ans et les adultes âgés de 18 à 34 ans ont déclaré un temps moyen de sédentarité par jour à l’école de 4,5 heures et de 5,2 heures respectivement. Les adultes de 18 à 34 ans et de 35 à 64 ans ont déclaré un temps moyen de sédentarité par jour au travail de 3,9 heures et de 4 heures respectivement. Le temps de sédentarité à l’école et au travail varie selon les sous-groupes. Chez les adultes de 18 à 34 ans, un temps de sédentarité plus élevé à l’école réduit la probabilité d’avoir une santé mentale « excellente/très bonne », tandis qu’un temps de sédentarité plus élevé au travail est associé à une plus grande probabilité de déclarer une santé générale « excellente/très bonne ».

**Conclusion:**

Les jeunes Canadiens et les adultes en âge de travailler déclarent un temps de sédentarité moyen de 4 à 5 heures par jour à l’école ou au travail. Il s’agit de la première étude qui estime le temps de sédentarité à l’école et au travail dans un échantillon représentatif de Canadiens. Elle contribuera à mieux faire connaître les comportements spécifiques sur la question, afin de mieux guider les interventions ciblées, notamment la lutte contre les inégalités en matière de sédentarité.

## Introduction

Sedentary behaviour is highly pervasive in daily life representing a suite of behaviours undertaken while in a sitting, lying, or reclining posture with a very low energy expenditure (≤ 1.5 METs) (Tremblay et al., 2017). Greater levels of sedentary time (ST) are associated with poor physical (e.g., chronic conditions, obesity, cardiovascular disease, diabetes, some cancers) and mental health (e.g., depression, quality of life, cognitive function) outcomes in children (Carson et al., 2016; Stiglic & Viner, 2019) and adults (Biswas et al., 2015; Saunders et al., 2020). In recognition of the benefits of lower ST alongside adequate physical activity and sleep, Canada developed 24-Hour Movement Guidelines (24-H Guidelines) for children and youth (5–17 years), adults (18–64 years), and older adults (≥ 65 years) (Ross et al., 2020; Tremblay et al., 2016). The 24-H Guidelines recommend that children and youth do not exceed 2 h/day of leisure screen time and limit sitting for extended periods, while adults do not exceed 8 h/day of total ST including 3 h/day of leisure screen time and breaking up long periods of sitting as often as possible (Canadian Society for Exercise Physiology, 2021).

Data from the 2018–2019 Canadian Health Measures Survey (CHMS) estimate that Canadian children and youth (5–17 years) are sedentary for 8.4 h/day and only 53.3% meet the leisure screen time recommendation (≤ 2 h/day). On average, adults (18 + years) are sedentary for 9.6 h/day and only 30.2% meet the sedentary behaviour recommendation of less than 8 h/day (Centre for Surveillance and Applied Research, 2023). Canadian health surveys have traditionally collected information on total ST (largely device-measured) and leisure time spent reading and using electronic devices (Prince et al. 2020b). Trend analyses suggested that accelerometer-measured ST has remained relatively stable over the last 10 + years, while self-reported leisure screen time, specifically electronic device use, has likely risen alongside declines in reading and television watching (Prince et al., 2020b). This suggests that the way in which Canadians are sedentary has changed. However, there is a lack of Canadian data sources for estimating ST in different domains such as work- and school-based sedentary behaviour.

Like physical activity, sedentary behaviour occurs in distinct domains that take place in specific settings (e.g., school, work, leisure, transportation). A meta-analysis of studies from the United States found that on average, youth spent 63% of their time sedentary while at school (Egan et al., 2019). Given the high proportion, international school-related sedentary behaviour recommendations for children and youth were recently released. These recommendations suggest to replace sedentary learning activities with movement-based learning when possible to support students’ health and well-being (Saunders et al., 2022).

Findings from a meta-analysis suggest that adults spent approximately 5 h/day or 60% of work time being sedentary, with office workers spending a significantly greater proportion of work time being sedentary compared to other occupations (72.5% vs. 49.7%) (Prince et al., 2019). Very few international surveillance systems report on daily amounts of time spent sitting at work. Data from the China National Nutrition and Health Survey in 2012 found that Chinese adults spent approximately 4 h/day in work-based sedentary behaviour (Ding et al., 2020). The 2014–2015 Australian Health Surveys found that 51% of full-time employed adults reported spending 4 or more h/day sitting while at work (Loyen et al., 2018). Similarly, a large Danish population survey found that working adults reported 4.4 h/day of work-based sitting (Aadahl et al., 2013).

Reporting on school- and work-related ST helps to increase awareness of these setting-specific behaviours alongside the already recognized leisure screen time recommendation. Additionally, given that Canadians spend a large portion of their days in these settings, they may be important targets for reducing non-leisure ST. In Canada, the Public Health Agency of Canada’s (PHAC) conceptual framework for the surveillance of Physical Activity, Sedentary behaviour and Sleep (PASS) recognizes the need for distinct surveillance of sedentary behaviour and its correlates and determinants, separate from light and higher intensities of physical activity, while also recognizing the need for a domain approach (i.e., ST in different settings) (Butler et al., 2019). Until recently, school and work ST data were not available for reporting. Thus, our objectives were to: estimate the daily amount of time Canadian youth and adults spent being sedentary while at school or work; examine whether daily school and work ST differ by sociodemographic characteristics; examine whether daily school and work ST differ between those meeting and those not meeting the aerobic physical activity, muscle strengthening, leisure screen time, and sleep recommendations from the 24-H Guidelines; and examine associations between school and work ST and indicators of health.

## Methods

### Data source

We used data from the 2020 Canadian Community Health Survey (CCHS) (share file), collected between January and March 2020, prior to the COVID-19 pandemic. The CCHS is an ongoing, annual cross-sectional survey conducted by Statistics Canada that collects self-reported health information from a representative sample of the Canadian household-dwelling population aged 12 and older living in the provinces and territories. It excludes individuals living on reserves and Crown Lands, institutionalized residents, full-time members of the Canadian forces, youth aged 12–17 living in foster care, and residents in certain remote regions. The CCHS covers approximately 98% of the Canadian population aged 12 years and older. In 2020, PHAC funded the Healthy Living Rapid Response (HLV-RR) within the CCHS to collect information on a variety of health behaviours, including sleep and muscle/bone strengthening activities, and a new question assessing daily duration of time spent sitting/lying/reclining while at school or work. The HLV-RR data were collected between January and March 2020 prior to the COVID-19 pandemic. The HLV-RR was asked to the same population of the CCHS except it excluded respondents living in the three territories and proxy respondents. In total, the HLV-RR was completed by 10,775 non-proxy respondents.

This study focused on school and working-aged (12–64 years) Canadians, who reported working and/or being in school during the previous week. From the 10,775 HLV-RR respondents, 3341 were excluded due to age ≥ 65 years (only 10.9% of this group reported working in the past week as their main activity), 2041 were excluded due to not having worked in a paid job or being in school during the previous week, and 151 were excluded for exceeding 10 or 12 h of school or work ST, respectively. A total sample of 5242 respondents were used for this analysis.

### School- and work-based sitting time

Respondents were asked: in the previous 7 days, while they were at school or work, how much time per day did they usually spend sitting, reclining, or lying down. They were asked to respond in continuous values of hours and minutes/day. To discern whether ST was likely reported for work or school, responses were combined with a question concerning the respondents’ main activity in the last week, which included options of paid work or going to school. In youth 12–15 years, the previous week’s main activity was automatically set to school.

We examined daily duration of time spent sedentary at school and at work. Time spent sedentary at school and work was capped at a maximum of 10 and 12 h/day, respectively. The daily maximum was selected based on an examination of outliers using a criterion of 3 standard deviations above the mean, which represented approximately 3% of the sample.

Only school ST was examined in youth 12–17 years as 97.6% of youth identified school as the main activity in the previous week. For adults 35–64 years, only work ST was examined as 98.5% of respondents identified work as their main activity during the past 7 days. Among adults 18–34 years, both school and work ST were examined as 33.3% identified school and 66.6% identified work as their main activity.

### Sociodemographic variables

We examined differences in school and work ST by age group (youth aged 12–17 years, adults aged 18–34 years and 35–64 years), sex (male, female), gender (men, women, gender diverse), cultural/racial background (non-racialized groups, racialized groups, Indigenous peoples), immigration status (landed immigrant, non-immigrant), highest level of respondent education (secondary graduation or less, post-secondary graduate), household income adequacy quintile (based on the adjusted ratio of total household income to low income corresponding to their household and community size), school status (identified by respondents as part-time or full-time, all respondents < 15 years of age automatically assigned full-time status), work status (full-time [≥ 30 h/week], part-time [< 30 h/week]), occupational group (based on the 2016 National Occupation Classification), residence location (urban or rural), living/family arrangement (living alone, in single-parent family, living with others or with other arrangements, in couple census family with children, in couple census family without children), and marital status (married/common law, widowed/divorced/separated/never married).

### Twenty-four-hour movement behaviours

We examined whether school or work ST differed by adherence to the age-specific aerobic physical activity recommendations (youth, ≥ 60 min/day; adults, ≥ 150 min/week of moderate-to-vigorous intensity physical activity), muscle and bone strengthening recommendations (youth, ≥ 3 times/week; adults, ≥ 2 times/week), leisure screen time recommendations (youth, ≤ 2 h/day; adults, ≤ 3 h/day), and sleep recommendations (youth 12–13 years, 9–11.99 h/night; youth 14–17 years, 8–10.99 h/night; adults, 7–9.99 h per night) of the 24-H Guidelines.

### Physical and mental health

We assessed whether school or work ST differed by self-reported measures of general and mental health, body mass index (BMI) category derived from self-reported height and weight (youth: based on age- and sex-specific BMI cut-points as defined by the World Health Organization (WHO); adults: corrected using the methods reported by Connor Gorber et al. (2008) and based on Health Canada and WHO body weight classification systems); and multimorbidity based on availability of reported conditions (diagnosis of 2 + of asthma, arthritis, cancer, diabetes, ischemic heart disease, stroke, chronic obstructive pulmonary disease, anxiety, and/or mood disorders) (Roberts et al., 2015).

### Statistical analyses

All analyses were conducted using SAS Enterprise Guide v.7.1 (SAS, Inc., Cary, NC). Descriptive statistics including average hours per day and 95% confidence intervals (CIs) are presented for time spent sedentary at school and work and by age group. Average hours per day spent sedentary at school or work were compared between sociodemographic groups, those meeting the individual 24-H Guideline recommendations, and physical and mental health status. Independent samples *t*-tests with post hoc comparisons using a Bonferroni correction were used to test for significant differences between groups. Associations with health indicators were examined using logistic regression adjusting for age, sex, income, and adherence to the aerobic physical activity recommendation.

All analyses were weighted using the rapid response survey weights provided by Statistics Canada. To account for survey design effects, 95% CIs were estimated using the bootstrap balanced repeated replication technique with 1000 replicate weights. Statistical significance was set at *p* < 0.05.

## Results

Youth reported an average of 4.5 (95% CI, 4.3–4.7) h/day of school ST. Adults 18–34 years reported an average of 5.2 (95% CI, 4.8–5.7) h/day of school ST and 3.9 (95% CI, 3.6–4.2) h/day of work ST. Adults 35–64 years reported an average of 4.0 (95% CI, 3.8–4.2) h/day of work ST.

### Differences in school and work sedentary time by sociodemographics

**Fig. 1:**
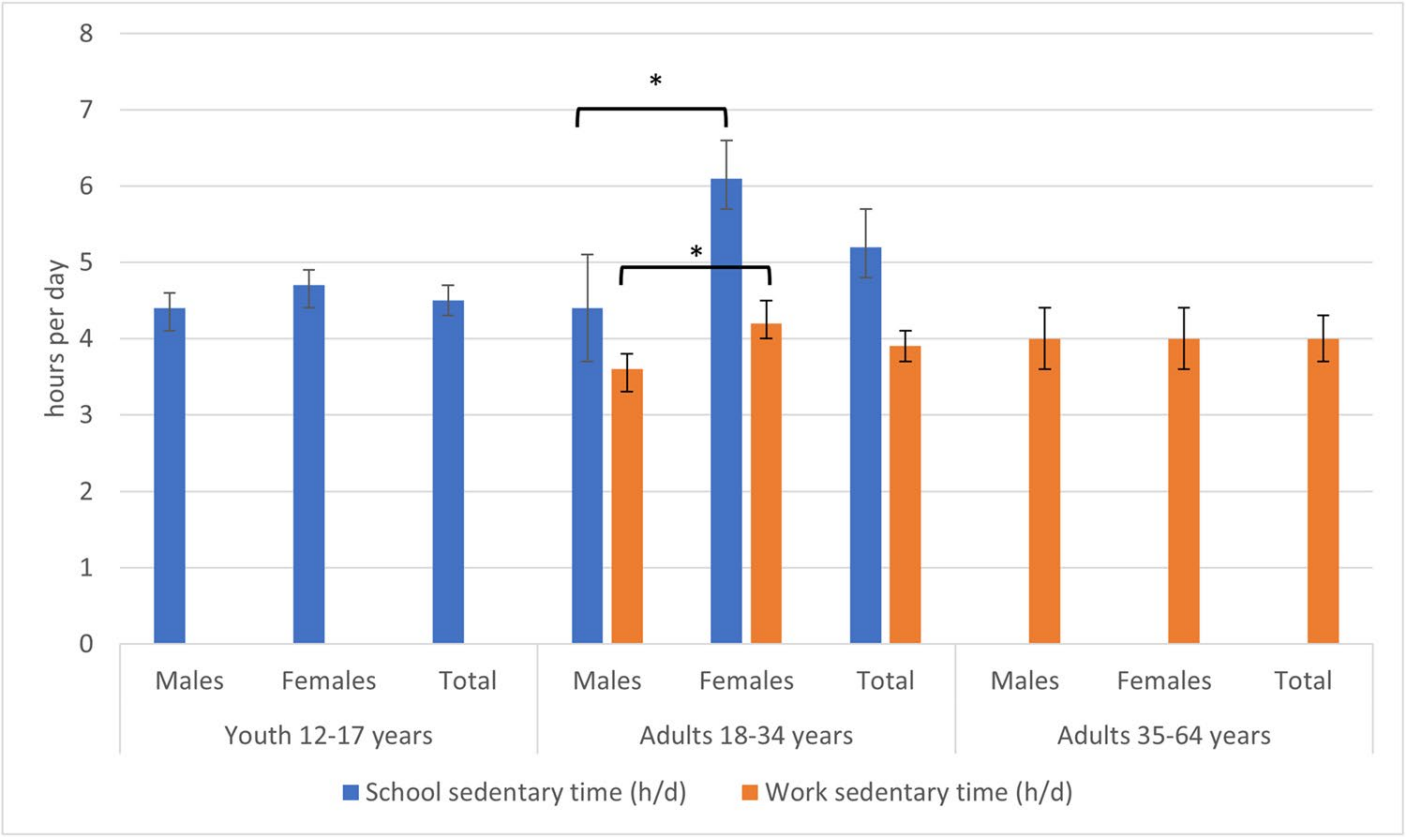
Sedentary time at school and work overall and in males and females, Canada excluding territories, 2020. *Significantly different between groups, *p* < 0.05. h/d, hours per day

Table 1 describes sociodemographic differences in school and work ST. Among youth, school ST did not differ across sociodemographic groups. Figure 1 shows school and work ST overall and by sex. Among adults 18–34 years, school ST was higher among females than among males (6.1 vs. 4.4 h/day). In this group, work ST was also higher among females than among males (4.2 vs. 3.6 h/day), and higher in urban vs. rural dwellers (4.0 vs. 3.1 h/day), those living alone vs. in a census couple with children (4.8 vs. 3.5 h/day), those with post-secondary vs. secondary school or less (4.3 vs. 2.5 h/day), those in part- vs. full-time school (4.8 vs. 3.3 h/day), those working full- vs. part-time (4.1 vs. 2.3 h/day), and by occupation group. In adults 35–64 years, work ST was higher in non-immigrants vs. landed immigrants (4.1 vs. 3.5 h/day), non-racialized groups vs. Indigenous peoples (4.1 vs. 3.1 h/day), those with post-secondary vs. secondary or less school (4.2 vs. 3.2 h/day), those with higher vs. lower incomes, those working full- vs. part-time (4.1 vs. 2.6 h/day), and by occupation group. Occupations that are traditionally considered more physically active (e.g., sales and services, manufacturing and utilities) reported shorter durations of work ST than those in more traditionally desk-based/sedentary occupations (e.g., natural and applied sciences, business, finance, and administration). Disaggregations for gender were calculated, but too few respondents identified as ‘gender diverse’ to generate stable results.

**Table 1:** Sedentary time at school and work by sociodemographic groups, Canada excluding territories, 2020

Characteristics	Youth 12–17 years			Adults 18–34 years						Adults 35–64 years		
	School sedentary time (hours/day)						Work sedentary time (hours/day)					
	Mean	LCL	UCL	Mean	LCL	UCL	Mean	LCL	UCL	Mean	LCL	UCL
Total	4.5	4.3	4.7	5.2	4.8	5.7	3.9	3.6	4.2	4.0	3.8	4.2
Sex												
Male	4.4	4.1	4.6	4.4*	3.7	5.1	3.6*	3.2	4.0	4.0	3.7	4.2
Female	4.7	4.4	4.9	6.1*	5.7	6.6	4.2*	3.8	4.6	4.0	3.8	4.3
Marital status												
Married or common law	N/A	.	.	5.8	4.6	6.9	4.0	3.6	4.4	4.0	3.8	4.2
Single^a^	N/A	.	.	5.2	4.7	5.7	3.8	3.4	4.2	4.0	3.7	4.3
Immigration status												
Landed immigrant	4.2	3.5	4.8	5.3	4.3	6.4	3.9	3.1	4.8	3.5*	3.1	4.0
Non-immigrant	4.6	4.4	4.8	5.2	4.7	5.7	3.9	3.6	4.2	4.1*	3.9	4.3
Cultural/racial background												
Racialized groups	4.2	3.9	4.6	5.3	4.6	6.0	4.2	3.6	4.9	3.8	3.3	4.3
Non-racialized groups	4.6	4.4	4.9	5.4	4.9	5.9	3.8	3.4	4.1	4.1*	3.9	4.3
Indigenous people	4.4	3.8	5.0	5.7	4.2	7.1	3.3	2.2	4.4	3.1*	2.3	3.9
Urban/rural status												
Urban	4.6	4.4	4.8	5.2	4.7	5.7	4.0*	3.7	4.3	4.0	3.8	4.2
Rural	4.2	3.9	4.5	5.0	4.1	6.0	3.1*	2.4	3.8	3.9	3.6	4.2
Living/family arrangement of respondent												
Living alone	F	.	.	5.6	4.7	6.5	4.8*	4.2	5.4	3.8	3.5	4.1
In single-parent family	4.9	4.6	5.3	4.9	4.0	5.8	3.4	2.6	4.2	4.2	3.6	4.8
Living with others or with other arrangements	4.6	4.0	5.2	5.1	4.0	6.2	3.6	2.9	4.3	3.8	3.1	4.4
In couple census family with children	4.4	4.2	4.6	5.3	4.6	6.1	3.5*	3.0	4.1	4.1	3.8	4.3
In couple census family without children	4.5	4.3	4.7	5.8	4.8	6.8	4.3	3.7	5.0	3.9	3.6	4.3
Education level												
Secondary graduate or less	N/A	.	.	5.1	4.6	5.7	2.5*	2.0	3.1	3.2*	2.8	3.5
Post-secondary graduate	N/A	.	.	5.4	4.4	6.5	4.3*	4.0	4.7	4.2*	4.0	4.4
Household income adequacy quintile												
Q1 (lowest)	4.5	4.1	4.9	5.4	4.7	6.0	3.9	3.1	4.7	2.8^b^	2.3	3.2
Q2	4.7	4.3	5.1	5.3	4.5	6.2	3.7	3.1	4.3	3.3^c^	2.9	3.7
Q3	4.4	4.0	4.8	4.8	3.6	6.0	3.3^d^	2.7	3.9	3.8^d^	3.5	4.2
Q4	4.5	4.0	4.0	5.2E	3.5	7.0	3.6	2.9	4.4	4.3	4.0	4.6
Q5 (highest)	4.6	4.3	4.9	5.2	4.2	6.2	4.9	4.3	5.5	4.7	4.3	5.0
School status												
Full-time	4.5	4.3	4.7	5.2	4.7	5.7	3.3E*	2.1	4.4	4.6E	2.9	6.3
Part-time	4.9	4.4	5.5	5.4	4.0	6.9	4.8*	3.8	5.9	4.9	3.6	6.1
Work status												
Full-time	F	.	.	5.5	3.8	7.2	4.1*	3.8	4.5	4.1*	3.9	4.3
Part-time	4.9	4.4	5.3	4.6	3.9	5.3	2.3*	1.6	3.0	2.6*	2.2	3.0
Occupation group												
Sales and service	N/A	.	.	N/A	.	.	2.2	1.7	2.8	2.5	2.2	2.8
Manufacturing and utilities	N/A	.	.	N/A	.	.	2.3E	0.9	3.7	2.6	1.8	3.4
Trades, transport, and equipment operators and related	N/A	.	.	N/A	.	.	2.6	1.9	3.4	3.3	2.8	3.9
Natural resources, agriculture, and related production	N/A	.	.	N/A	.	.	2.9E	1.5	4.4	3.5E	2.3	4.6
Health	N/A	.	.	N/A	.	.	3.5	2.8	4.1	3.1	2.6	3.6
Education, law, and social community and government services	N/A	.	.	N/A	.	.	3.7	2.7	4.7	3.3	2.9	3.7
Management	N/A	.	.	N/A	.	.	3.1E	1.5	4.6	4.2^j^	3.5	4.8
Art, culture, recreation, and sport	N/A	.	.	N/A	.	.	5.8^e^	4.6	7.0	4.9^ h^	4.1	5.8
Business, finance, and administration	N/A	.	.	N/A	.	.	6.1^f^	5.4	6.7	5.6^i^	5.2	5.9
Natural and applied sciences and related	N/A	.	.	N/A	.	.	6.4^ g^	5.8	6.9	5.8^ g^	5.4	6.3

Data source: Canadian Community Health Survey – Healthy Living Rapid Response Module, 2020

Abbreviations: *LCL*, lower confidence limit; *N/A*, not applicable; *UCL*, upper confidence limit

E—Interpret estimate with caution due to high sampling variability

F—Estimate is too unreliable to be released

^*^Significantly different between groups, *p* < 0.05

^a^Widowed, divorced, separated, or never married

^b^Income adequacy quintile 1 is significantly different from quintiles 3, 4, and 5, *p* < 0.05

^c^Income adequacy quintile 2 is significantly different from quintiles 4 and 5, *p* < 0.05

^d^Income adequacy quintile 3 is significantly different from quintile 5, *p* < 0.05

^e^Art, culture, recreation, and sport significantly different from ‘health’, ‘sales and services’, ‘manufacturing and utilities’, and ‘trades, transport, equipment operators and related’ *p* < 0.05

^f^Business, finance, and administration significantly different from ‘education, law, social/community/gov services’, ‘health’, ‘management’, ‘manufacturing and utilities’, ‘natural resources, agriculture and related’, ‘sales and service’, and ‘trades, transport, equipment operators and related’, *p* < 0.05

^g^Natural and applied sciences and related significantly different from ‘health’, ‘education, law, social/community/gov services’, ‘natural resources, agriculture and related’, ‘trades, transport, equipment operators and related’, ‘manufacturing and utilities’, and ‘sales and service’, *p* < 0.05

^h^Art, culture, recreation, and sport significantly different from ‘education, law, social/community/gov services’, ‘health’, ‘manufacturing and utilities’, and ‘sales and service’, *p* < 0.05

^i^Business, finance, and administration significantly different from ‘education, law, social/community/gov services’, ‘health’, ‘manufacturing and utilities’, ‘sales and service’, and ‘trades, transport, equipment operators and related’, *p* < 0.05

^j^‘Management’ significantly different from ‘natural and applied sciences’, ‘sales and service’, and ‘natural resources, agriculture and related’, *p* < 0.05

### Differences in sedentary time at school and work by 24-H Guideline adherence

**Fig. 2:**
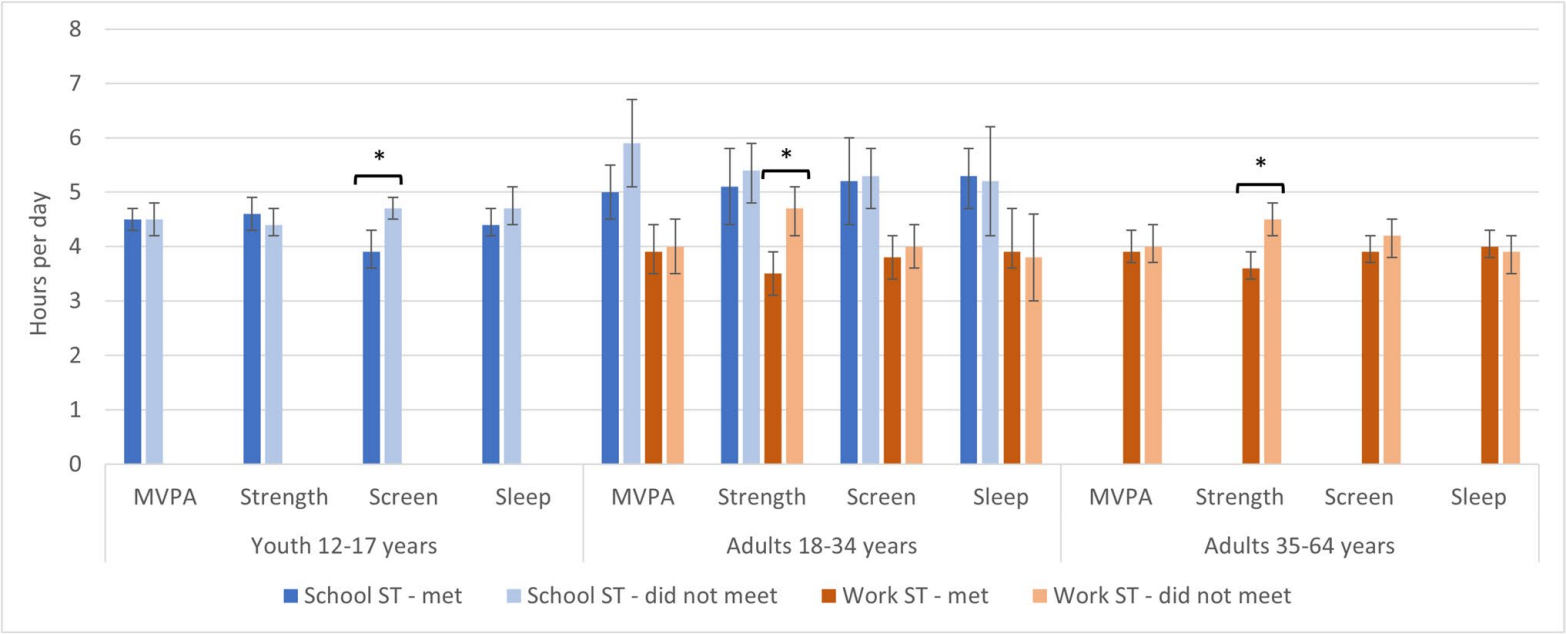
Sedentary time at school and work by adherence to 24-H Movement Guideline recommendations, Canada excluding territories, 2020. *Significantly different between groups, *p* < 0.05. MVPA, moderate-to-vigorous intensity physical activity; ST, sedentary time

Figure 2 and Supplementary Table 1 describe school and work ST by adherence to the individual 24-H Guideline recommendations (i.e., aerobic physical activity, muscle strengthening, leisure screen time, sleep). The only significant between-group difference for school ST was observed among youth for leisure screen time adherence, whereby those who did not adhere to the recommendations reported greater duration of school ST than those who met the recommendation (4.7 vs. 3.9 h/day, *p* = 0.0001). In adults, work ST was significantly higher among those who did not meet compared to those who met the muscle strengthening recommendation (18–34 years, 4.7 vs. 3.5 h/day, *p* = 0.0007; 35–64 years, 4.5 vs. 3.6 h/day, *p* < 0.001).

### Differences in sedentary time at school and work by health indicator

**Fig. 3:**
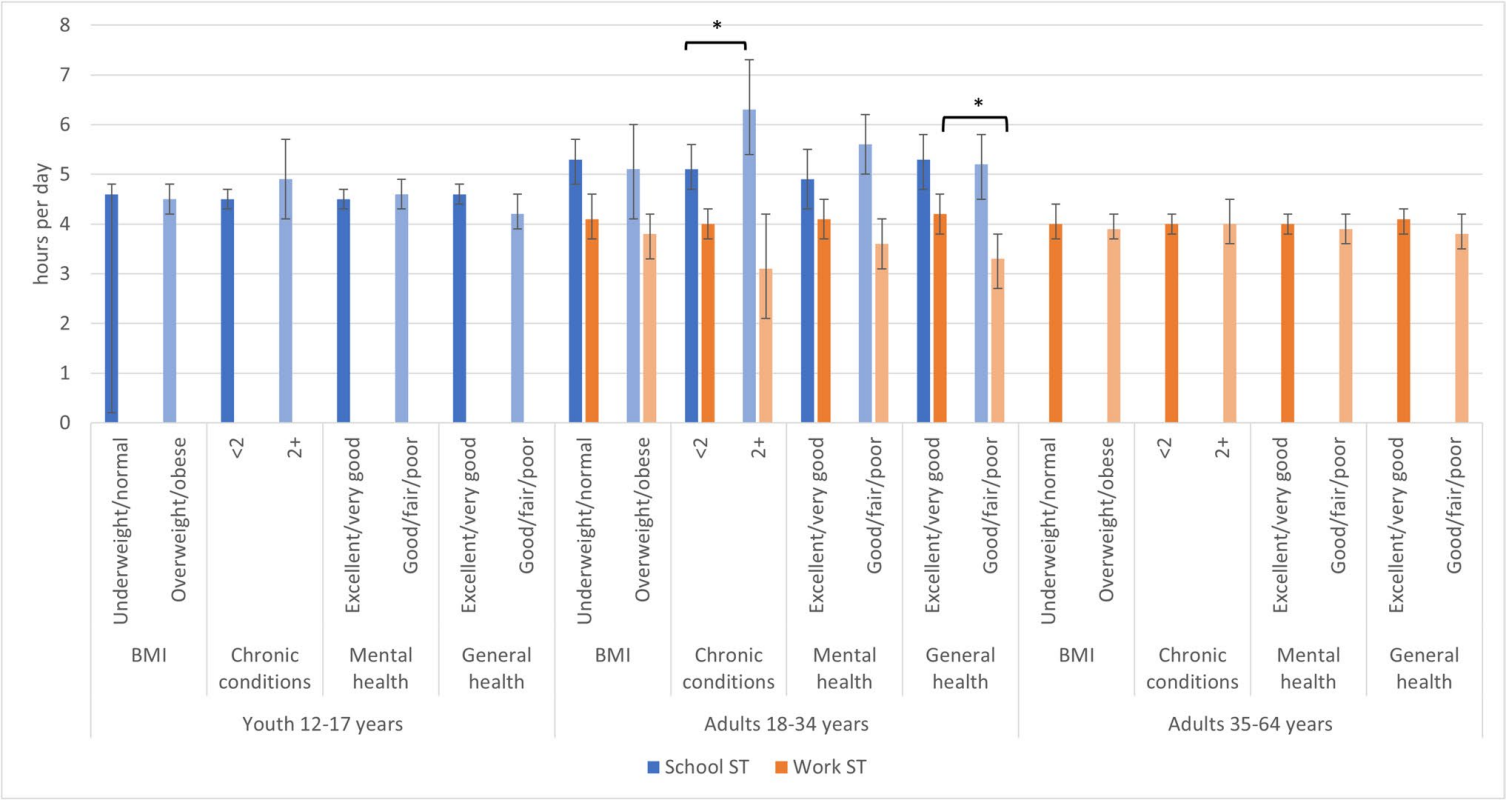
Sedentary time at school and work by health indicator, Canada excluding territories, 2020. *Significantly different between groups, *p* < 0.05. BMI, body mass index; ST, sedentary time

Figure 3 and Supplementary Table 2 describe school and work ST by health indicator. No differences in school or work ST were observed by BMI status or self-reported mental health. Adults 18–34 years who reported 2 or more chronic conditions reported more school ST than those who reported fewer than 2 chronic conditions (6.3 vs. 5.1 h/day).

Results of the multivariate logistic regressions found that after adjustment for age, sex, income, aerobic physical activity, and leisure screen time, there was no statistically significant association between school ST and the likelihood of 2 or more chronic conditions (aOR = 1.22; 95% CI, 0.89–1.66). Among adults 18–34 years, for every additional 1 h of school ST, the odds of ‘excellent or very good’ mental health are lower (aOR = 0.82; 95% CI, 0.68–0.997) than those for ‘good, fair or poor’ mental health, whereas for every additional 1 h of work ST, the odds of ‘excellent or very good’ general health were significantly higher than those for ‘good, fair or poor’ general health (aOR = 1.12; 95% CI, 1.04–1.21). Interestingly, in the adjusted model for work ST and general health, recreational screen time was associated with lower odds of ‘excellent or very good’ general health (aOR = 0.84; 95% CI, 0.74–0.96).

## Discussion

Using data from the 2020 CCHS HLV-RR, this study reports on the first national estimates for self-reported time spent sedentary at school and work among Canadians aged 12–64 years. Average school ST was generally higher than work ST. Only one demographic difference for school ST was noted: among adults 18–34 years, females reported more ST than males. Among adults 18–64 years, work ST was higher among those with post-secondary education vs. secondary or less, those with higher income adequacy, those in part-time vs. full-time school, and those in full-time vs. part-time work, and in some occupation groups (e.g., natural and applied sciences). Additionally, among adults 18–34 years, work ST was higher in females than in males, in urban vs. rural dwellers, and in those living alone vs. in a couple family with children. Among adults 35–64 years, work ST was also higher in non-immigrants vs. immigrants and in non-racialized groups vs. Indigenous peoples. Among youth, school ST was higher among those who did not meet the leisure screen time recommendation of the 24-H Guidelines. Among adults, work ST was significantly higher among those who did not adhere to the muscle strengthening recommendations. Additionally, among adults 18–34 years, greater school ST was associated with a lower likelihood of better mental health, whereas work ST was associated with a greater likelihood of better general health.

Currently, we are not aware of any other national-level data for school-based ST among youth or younger adults. This contrasts with a larger degree of reporting of population-level leisure screen time use among youth (Prince et al. 2020b). Youth reported an average of 4.5 h/day sedentary during school. This is not surprising given that youth attend school full-time in classrooms with predominantly desk-based learning. This represents a large portion of time spent sitting while at school (~ 65–70% of the day). Among youth, those who met the leisure screen time recommendation had lower levels of school ST. It is not clear why this difference was observed. It is possible that those who engaged in less time sitting at school (perhaps through other intramural activities and more movement-based breaks) are also less likely to engage in leisure screen time in lieu of more active pursuits during their leisure time. Further work is needed to understand whether a compensatory effect between school and leisure ST exists. Interestingly, unlike work ST, school ST does not differ by indicators of socioeconomic status (SES) and was not associated with indicators of self-reported health. This may be partially explained by the fact that most youth, regardless of SES, are exposed to similar school/classroom environments (~ 92% of Canadian youth attend publicly funded schools (Statistics Canada, 2021)). More research is needed to better understand whether school-based sedentary behaviour influences health. Research has shown that in contrast to screen-based sedentary behaviours, reading is associated with better health among children (Carson et al., 2016). It is, however, possible that greater ST at school correlates with more leisure ST as evidenced by the greater school ST among those who did not meet the leisure screen time recommendation, and greater leisure screen time use is associated with poorer health outcomes (Carson et al., 2016). School-based sedentary behaviour interventions have limited success with small but significant reductions in ST, with the greatest success to date seen with interventions that involve parents, behavioural change, and electronic TV monitoring devices (Biddle et al., 2014; dos Santos et al., 2019). Most interventions among children and adolescents have targeted the reduction of screen time (largely leisure-based) with education sessions and involvement of teachers and parents (Nguyen et al., 2020). Though limited in number, some school-based interventions have targeted school-time sedentary behaviour, with standing desks in classrooms showing promise for reducing ST (dos Santos et al., 2019). Emerging evidence suggests that in addition to generally reducing total ST, interventions could consider interrupting prolonged periods of ST by increasing sedentary behaviour ‘breaks’. Greater numbers of breaks and shorter bouts of ST have been associated with better markers of health in both youth (Saunders et al., 2013, 2018) and adults (Chastin et al., 2015; Saunders et al., 2018, 2020), though causal evidence is still needed to develop recommendations for break frequency and duration (Dempsey et al., 2020).

Among adults 18–34 years, women reported more school and work ST compared to men. Sex differences in ST have previously been reported elsewhere, often suggesting that women tend to engage in less leisure screen time than men (O’Donoghue et al., 2016; Prince et al., 2020c). Objective accelerometer data suggest that Canadian men and women from this age group do not differ in total daily ST. However, differences in leisure ST are often observed, whereby men in this age group tend to report higher leisure screen time whereas women report higher leisure reading time (Prince et al., 2020d). Systematic review evidence among university students has not generally observed gender differences in self-reported total daily sitting or screen time (Castro et al., 2020), but there are a lack of studies exploring school ST in this group. In Canada, women in full-time work spend a greater proportion of their day sedentary, and also tend to be more likely to work in occupations that are classified by lower levels of activity (Prince et al., 2020d).

Estimates from this study are similar to those observed in other surveillance studies with self-reported work-based ST in adults ranging from 4 to 5 h/day (Aadahl et al., 2013; Ding et al., 2020; Loyen et al., 2018). Like youth, estimates suggest that workers spend a large portion of their work time sedentary with an average of 4 h/day in adults aged 18–64 years. Several sociodemographic differences were observed including higher work ST among those of higher SES; this has been reported by other studies (O’Donoghue et al., 2016; Prince et al., 2017). Occupation (and occupational tasks) is often used as a measure of SES (Galobardes et al., 2006, 2007). Occupational differences were observed with workers in traditionally desk-oriented occupations reporting higher volumes of work ST (up to 6.4 h/day) compared to those occupations traditionally considered to be more physically active (as low as 2.2 h/day). This is consistent with other evidence (Loyen et al., 2016; Prince et al., 2019), as well as previous analyses using the CHMS which found that desk-based workers had significantly more device-measured total ST compared to those in more active occupations (Prince et al. 2020d). Interestingly, while total and work ST is known to differ by occupation, evidence suggests that physical activity remains low among most workers (Prince et al., 2020d).

Canada’s 24-H Guidelines recommend that working-age adults should limit total daily ST to 8 h/day (operationalized as 7 h/day via self-report, 9 h/day via device-measured (Ross et al., 2020)), limit leisure screen time to no more than 3 h/day, and break up long periods of sitting as often as possible. With an average of 4 h/day at work sedentary and an average of 3.2 h/day of leisure screen time (in adults 18–79 years) (Centre for Surveillance and Applied Research, 2023), most Canadian workers are likely exceeding these recommendations. While no international guidelines exist for work ST, in 2015, the United Kingdom published an expert statement on sedentary behaviour in office settings. The statement recommended that desk-based workers aim to progress towards accumulating 2 h/day of standing and light intensity activity (e.g., walking) during working hours, progressing to 4 h per full-time day (Buckley et al., 2015).

Most sedentary behaviour intervention work has focused on office settings, with interventions shown to effectively reduce ST by an average of 40 to 100 min per 8-h workday with those involving environmental changes, such as sit-stand desks and active permissive workstations, having greater success (Nguyen et al., 2020). Further work is needed to explore the reduction of workplace sitting in occupations outside of these settings (e.g., taxi drivers, bus operators) and across the socioeconomic spectrum, as it is unclear whether most office-based interventions have included workers of higher SES. This is important given the differences in work ST observed across socioeconomic categories. If interventions are not applied broadly across all workplaces/workers including those in lower socioeconomic jobs where they spend a large portion of their workday sitting, we risk widening the SES–health gap and increasing inequities.

Among adults 18–34 and 35–64 years, work ST was higher among those not meeting the muscle strengthening recommendation of 2 or more days per week. Mechanistically, it is difficult to know why this was observed. The question used to assess muscle strengthening activities on the CCHS could capture occupational activities such as carrying heavy loads which in turn are less likely to be observed in those whose occupation mainly requires sitting. Previous research has found that those whose work mainly involves sitting are more likely to meet the recommendation than those in heavy labour or physically demanding work. However, this is when muscle strengthening adherence is assessed more specifically by resistance training or strength exercises (Bennie et al., 2020).

Interestingly, in adults 18–34 years, higher volumes of work ST were associated with a greater likelihood of reporting ‘excellent or very good general’ health. Other studies have also found that while total ST and leisure ST are associated with poor health outcomes, work ST shows no or borderline significant associations (Chau et al., 2015; van Uffelen et al., 2010). The mechanism for this association is not clear, but it is important to observe this was only found in younger workers, where the majority (69%) report excellent or very good general health. Further work is needed to establish the directionality of this association. Additionally, it is possible that workers of higher SES have other positive health behaviours (e.g., greater time for leisure physical activity) which may be able to compensate for higher work ST.

### Strengths and limitations

The strengths of this study include the use of nationally representative data to report, for the first time, on self-reported school and work ST among Canadians, and compare ST across a variety of sociodemographic, movement, and health-related indicators. While the estimates for work ST are similar to those from other international studies, it is difficult to assess whether the school-based ST reflects what has been previously seen in other settings given the lack of measurement in this setting. It does, however, seem to align with device-measured ST during school from US studies. Self-reported sitting and ST, in general, tend to be under-reported when compared to device measures (Colley et al., 2022; Prince et al., 2020a). There is some evidence to suggest that anchoring reporting in specific subdivisions of the day (e.g., time at school or work) may help with recall accuracy (Dall et al., 2017). While this may have helped improve the recall of these behaviours, recall and response biases are likely still present. For the purposes of identifying ST as occurring during work or school, we used a combination of reported ST and the main reported activity from the previous week. While these recall periods are the same, it is possible that some respondents may be reporting a combination of full-time school or work with part-time work or school, therefore presenting the possibility of misclassification of some ST. The assessment of multimorbidity was based on availability of reported chronic conditions in the CCHS and, thus, may not represent all morbidity including back pain. This study is cross-sectional and, as such, we are unable to establish any causal linkage between school- and work-based ST and health outcomes. Finally, the data described herein were collected prior to the COVID-19 pandemic. The pandemic has likely impacted the school and work sedentary behaviour patterns of Canadians with a shift to virtual or hybrid arrangements. It will be important to reassess school and work ST in the future.

## Conclusion

This is the first time that school and work ST has been estimated in Canadians. Canadian youth and working-age adults report spending an average of 4–5 h/day sedentary at school and work, representing a large portion of time spent sedentary while in these settings. School-based ST was largely similar across sociodemographic groups, apart from being higher in female adults 18–64 years. In youth, school ST was higher among those who also did not meet the leisure screen time recommendation of the 24-H Guidelines. Work-based ST differed across several sociodemographic characteristics including socioeconomic status and occupation group. In adults 18–34 years, greater work ST was associated with better general health, whereas greater school ST was associated with poorer mental health. Further work is needed to understand the mechanisms and whether the association between higher work ST and better health in younger adults is causal or explained by residual confounding. Additionally, it is possible that workers of higher SES have other positive health behaviours which may be able to compensate for higher work ST.

The socioeconomic and demographic differences found in adults’ ST at work (e.g., females more than males, urban more than rural areas, living alone vs. in a couple with children, higher education vs. lower education, higher income vs. lower income) identify factors that might contribute to increased ST at work and add to the evidence base for policy and program development aimed at reducing ST. Future work is needed to better understand these inequalities in work ST and the potential role of school and work ST on health outcomes. Interventions targeting work ST should be considered, but with the recognition that they have the potential to widen the socioeconomic–health gap if not addressed across all workers whose occupations require sitting.

## Contributions to knowledge

What does this study add to existing knowledge?

- This is the first time that school- and work-based sedentary behaviours have been estimated at the national level in Canada.
- Canadian youth aged 12–17 years and adults aged 18–34 years report being sedentary for an average of 4.5 and 5.2 h/day at school, respectively.
- Canadian working-aged adults report an average of 4 h/day sedentary at work.
- Sedentary time at work differs by many sociodemographic characteristics including occupational group.
- Among adults 18–34 years, greater work sedentary time was associated with better self-reported general health, whereas greater school sedentary time was associated with poorer self-reported mental health.

What are the key implications for public health interventions, practice, or policy?

- Given that greater sedentary time is associated with poor physical and mental health and that Canadians spend a large portion of their days at school and work, understanding how much of this time is spent sedentary provides potential targets for intervention.
- Work sedentary time was shown to differ across sociodemographic groups, providing insight into inequalities in the exposure to sedentary time.
- Understanding inequalities in sedentary time is important for the design of future public health interventions.

### Supplementary Information

Below is the link to the electronic supplementary material.

Supplementary file1 (DOCX 20 KB)

## Data Availability

The datasets analyzed during the current study are available through the Research Data Centres (RDC) Program at Statistics Canada.
