# Supplementary material for: Sedentary time at school and work in Canada
Source: Can J Public Health. 2024 Jan 26;115(2):343–55. doi: 10.17269/s41997-023-00835-9 (PMC11006642; doi:10.17269/s41997-023-00835-9)
Supplement: Supplementary file 1 — Supplementary file1 (DOCX 20 KB) [file 41997_2023_835_MOESM1_ESM.docx]

Supplementary table 1. Sedentary time at school and work by adherence to 24-H Movement Guideline recommendations, Canada excluding territories, 2020

| **Guideline recommendations** | **Youth 12**–**17 years** | | | **Adults 18**–**34 years** | | | | | | **Adults 35**–**64 years** | | |
| --- | --- | --- | --- | --- | --- | --- | --- | --- | --- | --- | --- | --- |
|  | **School sedentary time (hours/day)** | | | | | | **Work sedentary time (hours/day)** | | | | | |
|  | **mean** | **LCL** | **UCL** | **mean** | **LCL** | **UCL** | **mean** | **LCL** | **UCL** | **mean** | **LCL** | **UCL** |
| **Moderate-to-vigorous intensity physical activity** | | | | | | | | | | | | |
| Met | 4.5 | 4.3 | 4.7 | 5.0 | 4.5 | 5.5 | 3.9 | 3.5 | 4.2 | 3.9 | 3.7 | 4.2 |
| Did not meet | 4.5 | 4.2 | 4.8 | 5.9 | 5.1 | 6.7 | 4.0 | 3.5 | 4.5 | 4.0 | 3.7 | 4.4 |
| **Muscle strengthening** | | | | | | | | | | | | |
| Met | 4.6 | 4.3 | 4.9 | 5.1 | 4.4 | 5.8 | **3.5*** | **3.1** | **3.9** | **3.6*** | **3.4** | **3.8** |
| Did not meet | 4.4 | 4.2 | 4.7 | 5.4 | 4.8 | 5.9 | **4.7*** | **4.2** | **5.1** | **4.5*** | **4.2** | **4.8** |
| **Leisure screen time** | | | | | | | | | | | | |
| Met | **3.9*** | **3.6** | **4.3** | 5.2 | 4.4 | 6.0 | 3.8 | 3.4 | 4.2 | 3.9 | 3.7 | 4.1 |
| Did not meet | **4.7*** | **4.5** | **4.9** | 5.3 | 4.7 | 5.8 | 4.0 | 3.5 | 4.5 | 4.2 | 3.8 | 4.5 |
| **Sleep** | | | | | | | | | | | | |
| Met | 4.4 | 4.2 | 4.7 | 5.3 | 4.7 | 5.8 | 3.9 | 3.6 | 4.3 | 4.0 | 3.8 | 4.2 |
| Did not meet | 4.7 | 4.4 | 5.1 | 5.2 | 4.2 | 6.2 | 3.8 | 3.0 | 4.6 | 3.9 | 3.5 | 4.2 |

Data source: Canadian Community Health Survey – Healthy Living Rapid Response Module, 2020.

Abbreviations: LCL, lower confidence limit; UCL, upper confidence limit.

*Significantly different between groups, *p* < 0.05.

Supplementary table 2. Sedentary time at school and work by health indicator, Canada excluding territories, 2020

| **Health indicator** | **Youth 12**–**17 years** | | | **Adults 18**–**34 years** | | | | | | **Adults 35**–**64 years** | | |
| --- | --- | --- | --- | --- | --- | --- | --- | --- | --- | --- | --- | --- |
|  | **School sedentary time (hours/day)** | | | | | | **Work sedentary time (hours/day)** | | | | | |
|  | **mean** | **LCL** | **UCL** | **mean** | **LCL** | **UCL** | **mean** | **LCL** | **UCL** | **mean** | **LCL** | **UCL** |
| **Body mass index** | | | | | | | | | | | | |
| Underweight or normal BMI | 4.6 | 4.4 | 4.8 | 5.3 | 4.8 | 5.7 | 4.1 | 3.7 | 4.6 | 4.0 | 3.7 | 4.4 |
| Overweight or obese BMI | 4.5 | 4.2 | 4.8 | 5.1 | 4.1 | 6.0 | 3.8 | 3.3 | 4.2 | 3.9 | 3.7 | 4.2 |
| **Chronic conditions** | | | | | | | | | | | | |
| < 2 chronic conditions | 4.5 | 4.3 | 4.7 | **5.1*** | **4.7** | **5.6** | 4.0 | 3.7 | 4.3 | 4.0 | 3.8 | 4.2 |
| 2 or more chronic conditions | 4.9 | 4.1 | 5.7 | **6.3*** | **5.4** | **7.3** | 3.1E | 2.1 | 4.2 | 4.0 | 3.6 | 4.5 |
| **Self-reported mental health** | | | | | | | | | | | | |
| Excellent or very good mental health | 4.5 | 4.3 | 4.7 | 4.9 | 4.3 | 5.5 | 4.1 | 3.7 | 4.5 | 4.0 | 3.8 | 4.2 |
| Good, fair or poor mental health | 4.6 | 4.3 | 4.9 | 5.6 | 5.0 | 6.3 | 3.6 | 3.1 | 4.1 | 3.9 | 3.6 | 4.2 |
| **Self-reported general health** | | | | | | | | | | | | |
| Excellent or very good general health | 4.6 | 4.4 | 4.8 | 5.3 | 4.7 | 5.8 | **4.2*** | **3.8** | **4.6** | 4.1 | 3.8 | 4.3 |
| Good, fair or poor general health | 4.2 | 3.9 | 4.6 | 5.2 | 4.5 | 5.8 | **3.3*** | **2.7** | **3.8** | 3.8 | 3.5 | 4.2 |

Data source: Canadian Community Health Survey – Healthy Living Rapid Response Module, 2020.

Abbreviations: BMI, body mass index; LCL, lower confidence limit; UCL, upper confidence limit.

*Significantly different between groups, *p* < 0.05.

E – Interpret estimate with caution due to high sampling variability.
